# G-Protein *α*-Subunit *Gsα* Is Required for Craniofacial Morphogenesis

**DOI:** 10.1371/journal.pone.0147535

**Published:** 2016-02-09

**Authors:** Run Lei, Ke Zhang, Yanxia Wei, Min Chen, Lee S. Weinstein, Yang Hong, Minyan Zhu, Hongchang Li, Huashun Li

**Affiliations:** 1 West China Developmental & Stem Cell Institute, West China Second Hospital, and State Key Laboratory of Biotherapy, West China Hospital, Sichuan University, Chengdu, Sichuan, China; 2 Shenzhen Key Laboratory for Molecular Biology of Neural Development, Laboratory of Developmental and Regenerative biology, Institute of Biomedicine & Biotechnology, Shenzhen Institutes of Advanced Technology, Chinese Academy of Sciences, Shenzhen, Guangdong, China; 3 SARITEX Center for Stem Cell Engineering Translational Medicine, Shanghai East Hospital, Tongji University School of Medicine, Chinese Academy of Sciences, Shanghai, China; 4 Metabolic Diseases Branch, National Institute of Diabetes and Digestive and Kidney Diseases, National Institutes of Health, Bethesda, Maryland, United States of America; 5 Department of Cell Biology & Physiology, University of Pittsburgh School of Medicine, Pittsburgh, Pennsylvania, United States of America; Medical University of South Carolina, UNITED STATES

## Abstract

The heterotrimeric G protein subunit Gsα couples receptors to activate adenylyl cyclase and is required for the intracellular cAMP response and protein kinase A (PKA) activation. Gsα is ubiquitously expressed in many cell types; however, the role of Gsα in neural crest cells (NCCs) remains unclear. Here we report that NCCs-specific *Gsα* knockout mice die within hours after birth and exhibit dramatic craniofacial malformations, including hypoplastic maxilla and mandible, cleft palate and craniofacial skeleton defects. Histological and anatomical analysis reveal that the cleft palate in *Gsα* knockout mice is a secondary defect resulting from craniofacial skeleton deficiencies. In *Gsα* knockout mice, the morphologies of NCCs-derived cranial nerves are normal, but the development of dorsal root and sympathetic ganglia are impaired. Furthermore, loss of *Gsα* in NCCs does not affect cranial NCCs migration or cell proliferation, but significantly accelerate osteochondrogenic differentiation. Taken together, our study suggests that Gsα is required for neural crest cells-derived craniofacial development.

## Introduction

Neural crest cells (NCCs) are transient population of multipotent progenitors which arise from the border between neural plate and epidermis. During neurulation, NCCs undergo an epithelium to mesenchyme transition (EMT) process, migrate stereotypically to different locations, then differentiate into multiple cell types [1,2,3,4,5,6,7]. Cranial NCCs, which originate from posterior forebrain and posterior hindbrain, contribute to bones, cartilages, connective tissues and cranial ganglia in face and neck. Cardiac NCCs, a subpopulation of cranial NCCs emanating from rhombomeres 6–8, give rise to parts of cardiac septum, thyroid and thymus. Trunk NCCs arising from caudal to the fourth somite are necessary for the formation of enteric peripheral nervous system (PNS), endocrine organs, pigment cells, as well as dorsal root ganglion (DRG) and sympathetic ganglion in PNS. In mammals, craniofacial morphogenesis requires accurate coordination of cranial NCCs migration, proliferation, apoptosis and differentiation. Disruption of these cellular programs would cause numerous congenital defects including craniofacial malformations, which comprise at least one-third of human birth defects [4,8]. Clefts of lip and/or palate (CLP) are the most common craniofacial defects, occurring approximately 1 in 700 neonates [9].

The α subunit of heterotrimeric G protein (Gsα) is encoded by *GNAS* (*Gnas* in mice), which is ubiquitously expressed in many cell types and responsible for receptor-stimulated cAMP generation and activation of protein kinase A (PKA) pathway [10,11]. *Gnas* homozygous mutation in mice causes embryonic lethality; *Gnas* heterozygous with the inheritance of maternal or paternal mutation exhibit distinct phenotypes including neurological abnormalities, lethality after birth and small with narrow bodies [12]. It has been shown that *Gsα* mutations cause skeletal disorders in humans and mice. Heterozygous loss-of-function mutations in *GNAS* lead to Albright hereditary osteodystrophy (AHO), which is characterized by short stature, brachydactyly, developmental delay or mental deficits, and facial defects such as orbital hypertelorism and depressed nasal bridge. In contrast, mutations activating *GNAS* result in McCune–Albright syndrome (MAS). The MAS patients exhibit fibrous dysplasia lesions, which is characterized by weakened osteoblast differentiation [11,13,14,15,16,17]. In mice, chondrocyte-specific ablation of *Gsα* leads to growth plate defects and hypertrophic differentiation of growth plate cartilages [18,19]. In mice osteoprogenitors, loss of *Gsα* signaling decreased the commitment of mesenchymal progenitors to osteoblast lineage and accelerated osteogenic differentiation [20]. In addition, Sakamoto and colleagues reported that the deletion of *Gsα* in differentiated osteoblast resulted in reduced trabecular bone volume, increased cortical bone thickness and abnormalities in craniofacial skeleton [21]. Together, these findings indicate that *Gsα* signaling is crucial for skeletal formation; however, its role in cranial NCCs-derived craniofacial skeletal development has not been investigated.

In the present study, we examined the potential function of *Gsα* in craniofacial development using *Wnt1-cre*-mediated *Gsα* knockout (KO) mice. NCCs-specific knockout *Gsα* results in respiratory distress, inability to suckle and postnatal death in mice. Histological examinations show that *Wnt1-cre;Gsα*^*f/f*^ mutant exhibits craniofacial skeletal defects and cleft palate, premature ossification within maxilla and mandible, nasal septum, hyoid and laryngeal cartilages, as well as impaired development of dorsal root and sympathetic ganglia. Further results reveal that the cleft palate phenotype in *Wnt1-cre;Gsα*^*f/f*^ mutant is a secondary defect caused by craniofacial skeletal deficiencies. Cellular function analysis shows that the cranial NCCs migration and cell proliferation are normal, but the osteochondrogenic differentiation is accelerated in *Wnt1-cre;Gsα*^*f/f*^ mutant. Altogether, these results demonstrate that Gsα plays a critical function in the development of cranial neural crest cells.

## Results

### Specific deletion of *Gsα* in NCCs leads to craniofacial malformations, defective development of dorsal root and sympathetic ganglia

**Fig 1:**
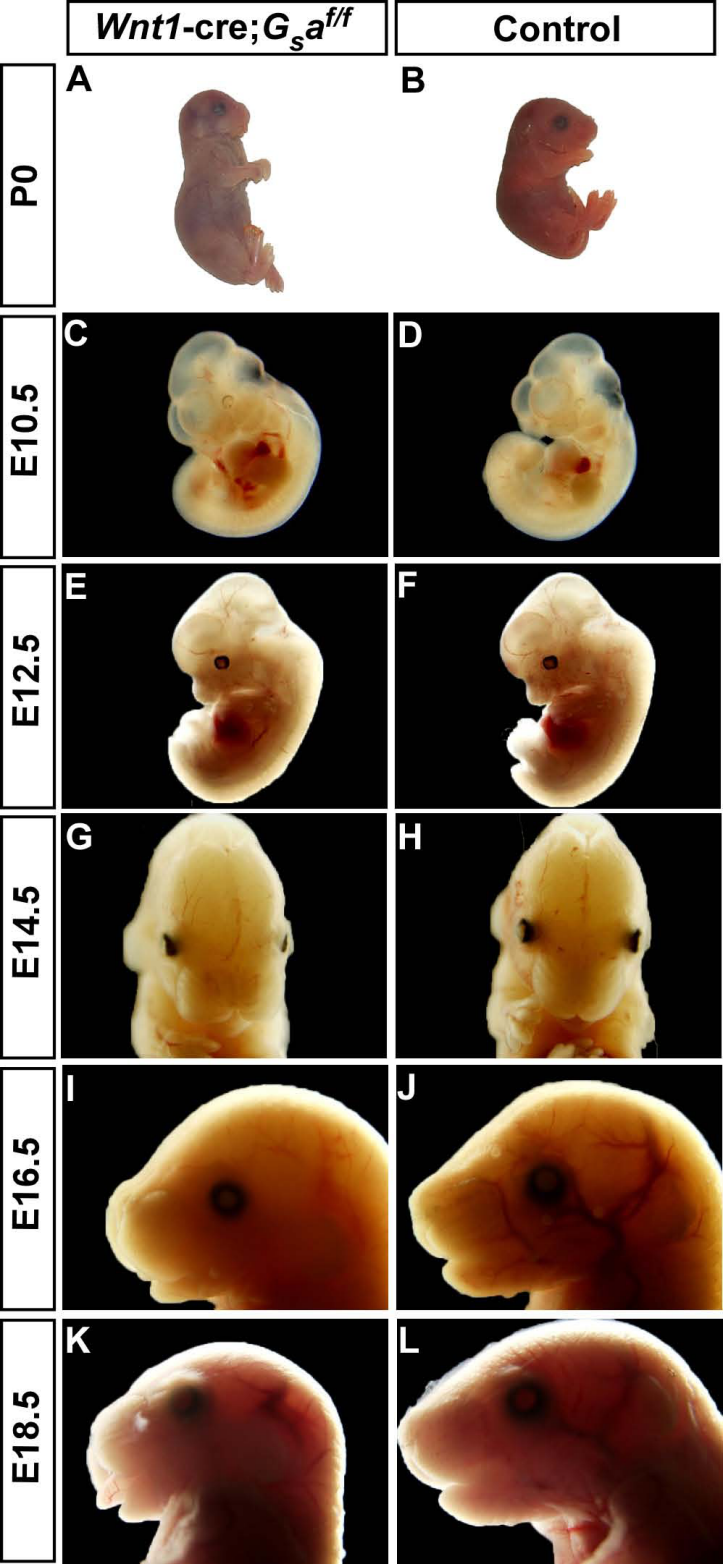
Loss of *Gsα* in NCCs results in severe craniofacial malformations. (A, B) P0 *Wnt1-cre;Gsα*^*f/f*^ mutant and control. *Wnt1-cre;Gsα*^*f/f*^ mutant mice become cyanotic and die within hours after birth, exhibit domed skull, shortened maxilla and mandible and exposed tongue. (C-F) The gross appearance of *Wnt1-cre;Gsα*^*f/f*^ mutants (C and E) and controls (D and F) are identical at E10.5 and E12.5 respectively. (G, H) E14.5 *Wnt1-cre;Gsα*^*f/f*^ mutant and control. *Wnt1-cre;Gsα*^*f/f*^ embryos show short snout, round face and hypertelorism. (I-L) E16.5 and E18.5 *Wnt1-cre;Gsα*^*f/f*^ mutants (I and K) display exposed tongues and shortened maxilla and mandible compare to controls (J and L).

To establish the requirement for *Gsα* in NCC-derived tissues, *Gsα*^*flox/flox*^ mice [22] were crossed with Wnt1-cre mice in which Cre recombinase is expressed in migrating NCCs [23]. *Wnt1-cre;Gsα*^*f/f*^ mutant mice were born at expected Mendelian ratios, but were unable to suckle and progressively became more cyanotic, and all neonates died within hours after birth. Prenatal lethality was not observed. All these mutants exhibited severe craniofacial abnormalities at postnatal day 0 (P0), including domed skull, shortened maxilla and mandible, and exposed tongue (Fig 1A). To define the progressive development process in *Wnt1-cre;Gsα*^*f/f*^ mutants, embryos from different stages were examined by anatomical analysis. At embryonic day 10.5 and 12.5 (E10.5 and E12.5), the gross morphologies of *Wnt1-cre;Gsα*^*f/f*^ mutants and controls were similar (Fig 1C–1F). However, the E14.5 *Wnt1-cre;Gsα*^*f/f*^ embryos displayed short snout, round face and orbital hypertelorism (Fig 1G). The craniofacial abnormalities were severer at later embryonic stages, as shown by the maxilla and mandible in E16.5 and E18.5 *Wnt1-cre;Gsα*^*f/f*^ embryos were dramatically shortened which leads to extended tongues (Fig 1I and 1K). Next, to determine the effect of *Gsα* on cranial nerves morphogenesis, E10.5 and E12.5 embryos were immunostained with anti-neurofilament antibody. The formation of cranial nerves was normal in E10.5 and E12.5 *Wnt1-cre;Gsα*^*f/f*^ embryos compared to controls (Fig A-D in S1 Fig). To analyze the effects of *Gsα* deletion in NCCs-derived cardiac, DRG and sympathetic development, we crossed *Gsα*^*flox/flox*^ mice with Rosa26-reporter transgenic mice to generate *Wnt1-cre;Gsα*^*f/f*^*;R26R* mutants. All these *Wnt1-cre;Gsα*^*f/f*^*;R26R* mutants also exhibited craniofacial malformations and cleft palates. Whole-mount X-gal staining results showed the gross morphologies of outflow tract and cardiac development were similar between E17.5 *Wnt1-cre;Gsα*^*f/f*^ and controls (Fig E and F in S1 Fig). The formation of DRG and sympathetic ganglia in E15.5 mutants were normal (Fig A and B in S2 Fig), but reduced size of DRG and sympathetic ganglia were observed in E16.5 and E17.5 mutants (Fig C-J in S2 Fig). Together, these results indicate that loss of *Gsα* in NCCs leads to craniofacial malformations, defective development of DRG and sympathetic ganglia.

### *Wnt1-cre;Gsα*^*f/f*^ mutants exhibit craniofacial skeletal defects

**Fig 2:**
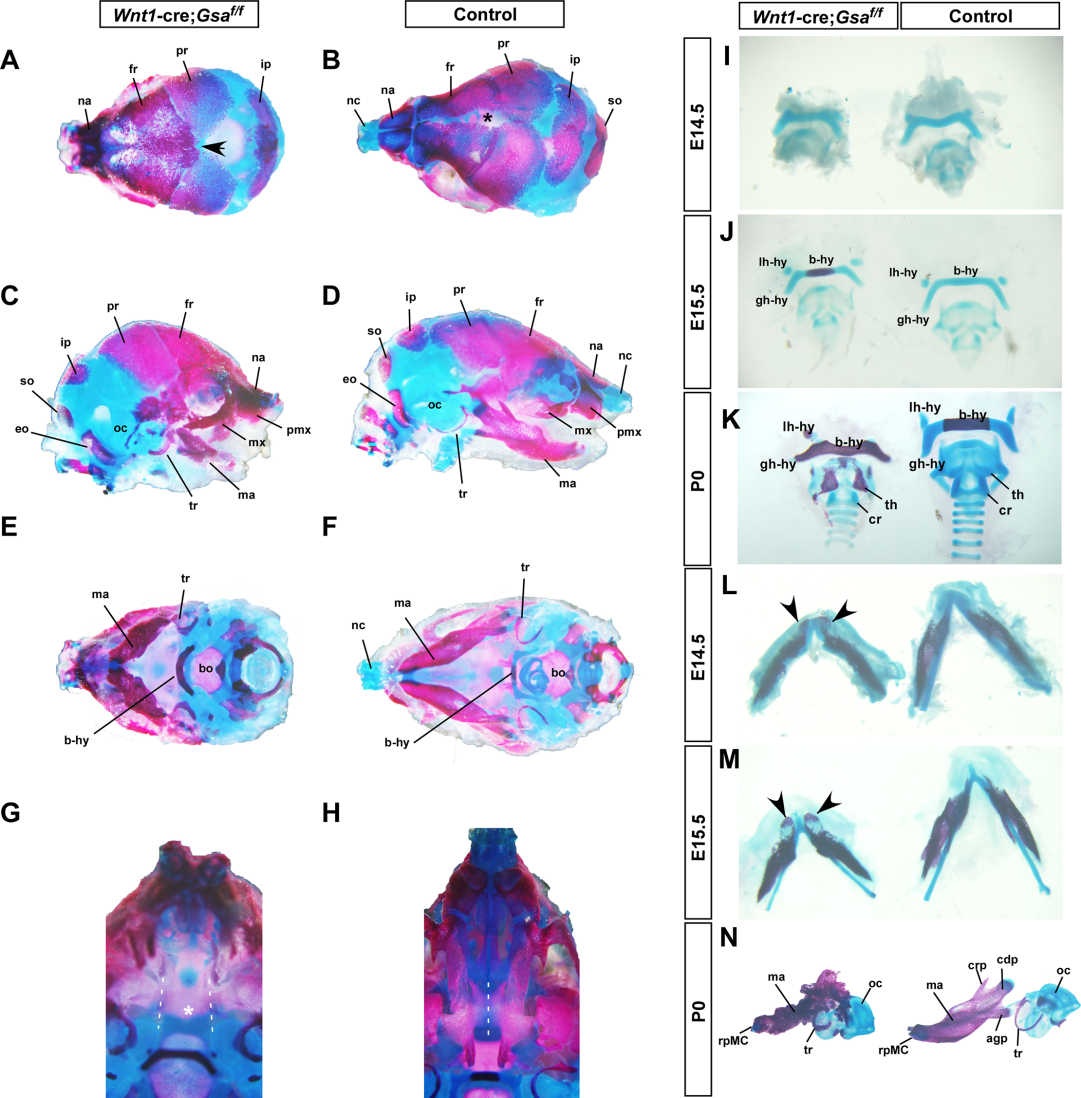
*Wnt1-cre;Gsα*^*f/f*^ mutants exhibit craniofacial skeleton defects. (A-F) Neonates skeleton preparations of newborn *Wnt1-cre;Gsα*^*f/f*^ mutant and control stained with alcian blue and alizarin red; dorsal (A, B), lateral (C, D) and ventral (E, F) views. A suture between frontal bones in control (asterisk in B), but craniosynostosis (A, arrowhead) in*Wnt1-cre;Gsα*^*f/f*^ mutant. (C-F) In *Wnt1-cre;Gsα*^*f/f*^ mutant, the premaxilla(pmx), maxilla (mx) and mandible (ma) are hypoplastic and deformed, the nasal capsule cartilage (nc) is missing, the body of hyoid bone (b-hy) is over-ossified, and the tympanic rings are thickened and deformed. (G, H) The mandible is removed to enhance the view of palatal bone. Palatal bones are fused to form the secondary palate in control (H, dashed line), but severely hypoplastic and cleft in *Wnt1-cre;Gsα*^*f/f*^ mutant (G, dashed lines and asterisk). (I-K) Dissected hyoid and laryngeal skeletons from E14.5 (I), E15.5 (J), and P0 (K) skeletal staining samples. *Wnt1-cre;Gsα*^*f/f*^ mutants exhibit premature ossification in the body of hyoid bone at E15.5, and abnormal ossification in hyoid bone and thyroid cartilage at P0. (L-N) Dissected mandibles from the same embryos shown in Fig 2 I-K. Arrowheads in (L) and (M) indicate the abnormal ossification of incisor tip in *Wnt1-cre;Gsα*^*f/f*^ mutants. The NCCs-derived mandible and tympanic rings are severely malformed, but the mesoderm-derived otic capsule is normal in P0 *Wnt1-cre;Gsα*^*f/f*^ mutant (N). agp, angular process; b-hy, body of the hyoid bone; bo, basioccipital; cdp, condylar process; crp, coronoid process; eo, exoccipital; fr, frontal; gh-hy, greater horn of the hyoid bone; ip, interparietal; lh-hy, lesser horn of the hyoid bone; ma, mandible; mx, maxilla; na, nasal bone; nc, nasal capsule; oc, otic capsule; pmx, premaxilla; pr, parietal; rpMC, rostral process of Meckel’s cartilage; so, supraoccipital; th, thyroid cartilage; tr, tympanic ring.

To analyze the effect of *Gsα* loss on craniofacial structures, skeletal preparation from newborn *Wnt1-cre;Gsα*^*f/f*^ mutants and controls were stained with Alcian Blue and Alizarin Red to reveal cartilages and bones, respectively. A suture between frontal bones was observed in control, but it was abnormally fused in *Wnt1-cre;Gsα*^*f/f*^ mutant (Fig 2A and 2B). In mutant, the cartilage of nasal capsule (nc) was absent, the maxilla(x), premaxilla (px) and mandible (ma) were hypoplastic and malformed (Fig 2C), and the tympanic ring (tr) and body of hyoid bone (b-hy) exhibited abnormal ossification (Fig 2E) compared to that in control (Fig 2D and 2F). Interestingly, the nasal capsule, maxilla, premaxilla, mandible, tympanic ring and body of hyoid bone, which are assigned NCCs originals, were absent or severely malformed, whereas the skeleton elements from mesoderm including parietal (pr), lateral portion of the interparietal (ip), supraoccipital (so), exoccipital (eo), basioccipital (bo) and otic capsule (oc), were formed normally in *Wnt1-cre;Gsα*^*f/f*^ mutant (Fig 2C–2F). The bilateral palatal bones extended horizontally and eventually fused to form palate in control, but they were hypoplastic and thus palate was cleft in mutant (Fig 2G and 2H).

Given that the hyoid bone and mandibular bone in P0 *Wnt1-cre;Gsα*^*f/f*^ mutant were heavily ossified (Fig 2E), to further define this defect, we observed the hyoid and laryngeal skeletons and mandible isolated from different stages. The appearance of laryngeal skeletons in *Wnt1-cre;Gsα*^*f/f*^ mutant were normal until E14.5 (Fig 2I), but starting from E15.5, the premature ossification in body of hyoid bone was observed (Fig 2J). This abnormal ossification in P0 *Wnt1-cre;Gsα*^*f/f*^ mutant was much more severe than that in control (Fig 2K). Additionally, dissected mandibles from the same E14.5 and E15.5 embryos shown in Fig 2I and 2J revealed that *Wnt1-cre;Gsα*^*f/f*^ mutants exhibited abnormal ossification in the incisor tip (Fig 2L and 2M). Moreover, the appearance of mandibular bone in P0 mutant was dramatically malformed; and the condylar, coronoid and angular processes (cdp, crp and agp) in mutant mandible were missing compared to that in control (Fig 2N).

Taken together, our data indicate that *Gsα* expression in NCCs is required for the development of craniofacial skeleton.

### Cleft palate in *Wnt1-cre;Gsα*^*f/f*^ mutant is caused by craniofacial skeleton defects

**Fig 3:**
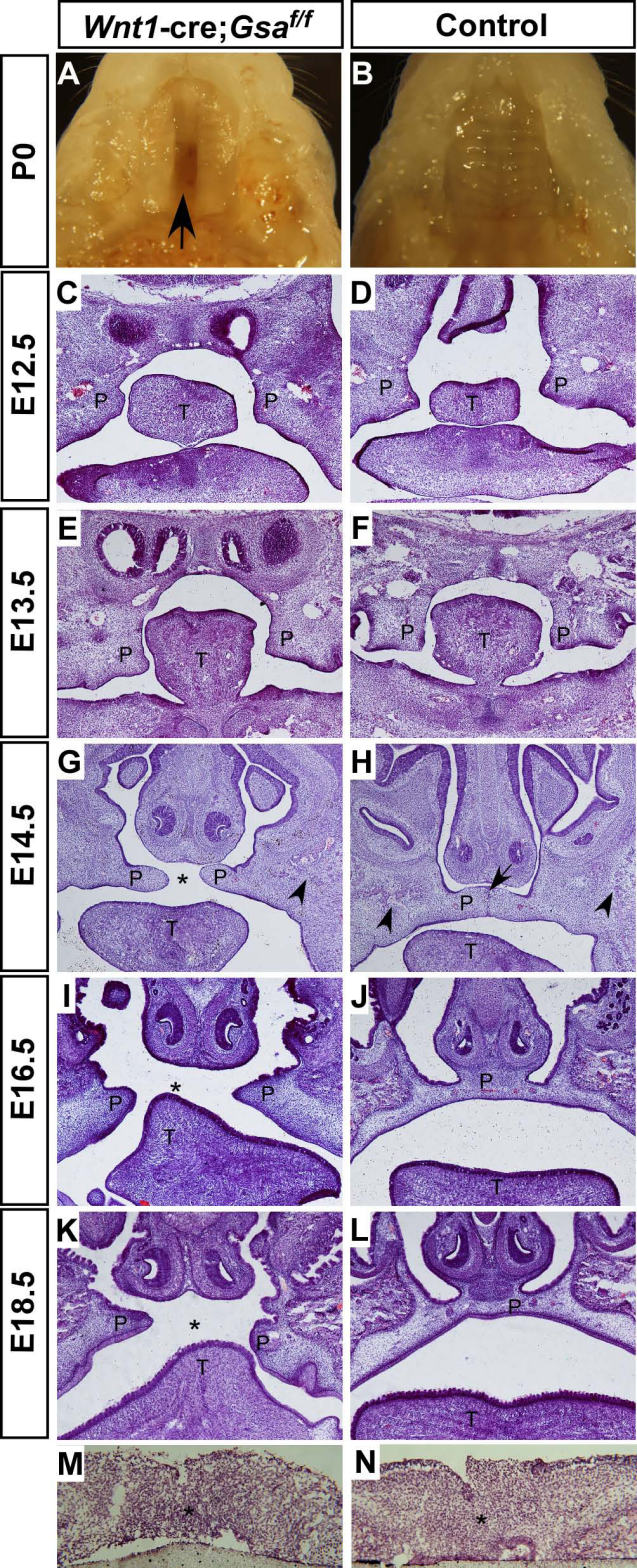
*Wnt1-cre;Gsα*^*f/f*^ mutants exhibit cleft palate. (A, B) Ventral view of palates after removal of mandible in P0 *Wnt1-cre;Gsα*^*f/f*^ mutant and control. Fused palate in control (B), but cleft palate in *Gsα* knockout mice (A, arrow). (C-H) H&E staining of head coronal sections in *Wnt1-cre;Gsα*^*f/f*^ mutants and controls from different embryonic stages. The palatal shelves grow vertically at two sides of tongue at E12.5 and E13.5 (C-F); and then the palatal shelves have been elevated horizontally above the tongue and fused with remnant medial edge epithelium in control at E14.5 (H, arrow), however they fail to fuse and a cleft was observed in *Wnt1-cre;Gsα*^*f/f*^ mutant (G, asterisk), the initiation of palatal bone formation are indicated by arrowheads in (G and H). (I-L) The palatal shelves have completely fused with flat tongues and disappearance of midline epithelium in E16.5 and E18.5 controls (J and L), in contrast to the cleft palates with arched tongues in *Wnt1-cre;Gsα*^*f/f*^ mutants (I and K). (M, N) *In vitro* organ culture shows *Wnt1-cre;Gsα*^*f/f*^ mutant palatal shelves are able to fuse, asterisk indicates the disappearance of midline epithelium. P, palate; T, tongue.

*Wnt1-cre;Gsα*^*f/f*^ mutants displayed non-fusion of palatal bones, therefore we removed mandible and found that the newborn *Wnt1-cre;Gsα*^*f/f*^ mutant had a complete cleft palate (Fig 3A). To determine the onset of cleft palate in mutant, palates from different embryonic stages were examined by histological staining. In controls, the palatal shelves arose from maxillary prominence at E12.5 (Fig 3D) and descended vertically down to the two sides of tongue at E13.5 (Fig 3F), then they elevated to the horizontal position above tongue and elongated to fuse with remnant medial edge epithelium (MEE) at E14.5 (Fig 3H). Finally, the control mice displayed fused palates with flat tongues (Fig 3J and 3L). In contrast, this progressive process was disrupted by the loss of *Gsα*. The palatal shelves in mutant were well developed and properly elevated to the horizontal position above tongue (Fig 3C, 3E and 3G), but they failed to elongate or fuse at E14.5 (Fig 3G), and consequently the E16.5 and E18.5 mutant exhibited cleft palates with protuberant tongues (Fig 3I and 3K).

The cleft palate phenotype in *Wnt1-cre;Gsα*^*f/f*^ mutant was tightly accompanied with dramatic craniofacial skeleton defects, therefore raising a question that whether the cleft palate resulted from a primary defect in palatal development or was secondary to craniofacial skeleton defects. To this end, we examined the fusion ability of palatal shelves by performing an *in vitro* palatal shelf organ culture experiment. In brief, each pair of palatal shelves was dissected from E13.5 embryos and placed on Millipore filters with touching. By 72 hours in culture, all palatal specimens from controls and mutants showed complete fusion with normal disappearance of MEE (Fig 3M and 3N), suggesting that palatal shelves in *Wnt1-cre;Gsα*^*f/f*^ mutants retain the ability to fuse. Given that *Wnt1*-cre-mediated deletion of *Gsα* occurs both in NCCs-derived craniofacial skeletons and palatal mesenchyme, to ablate *Gsα* in the mesenchyme and epithelium of palatal shelves but not in craniofacial skeletons, we generated *Nestin-cre;Gsα*^*f/f*^ mutants by crossbreeding *Gsα*^flox/flox^ mice with *Nestin-cre* mice [24]. Morphological analysis showed that the newborn *Nestin-cre;Gsα*^*f/f*^ mutants displayed normal craniofacial structures and completely fused palates (data not shown), suggesting that the deletion of *Gsα* in mesenchyme and epithelium of palatal shelves does not cause cleft palate. Collectively, these results strongly suggest that the cleft palate in *Wnt1-cre;Gsα*^*f/f*^ mutant is not caused by intrinsic defects within palate but most probably is secondary to craniofacial skeleton defects.

### Specific deletion of *Gsα* in NCCs does not affect CNCCs migration or cell proliferation

**Fig 4:**
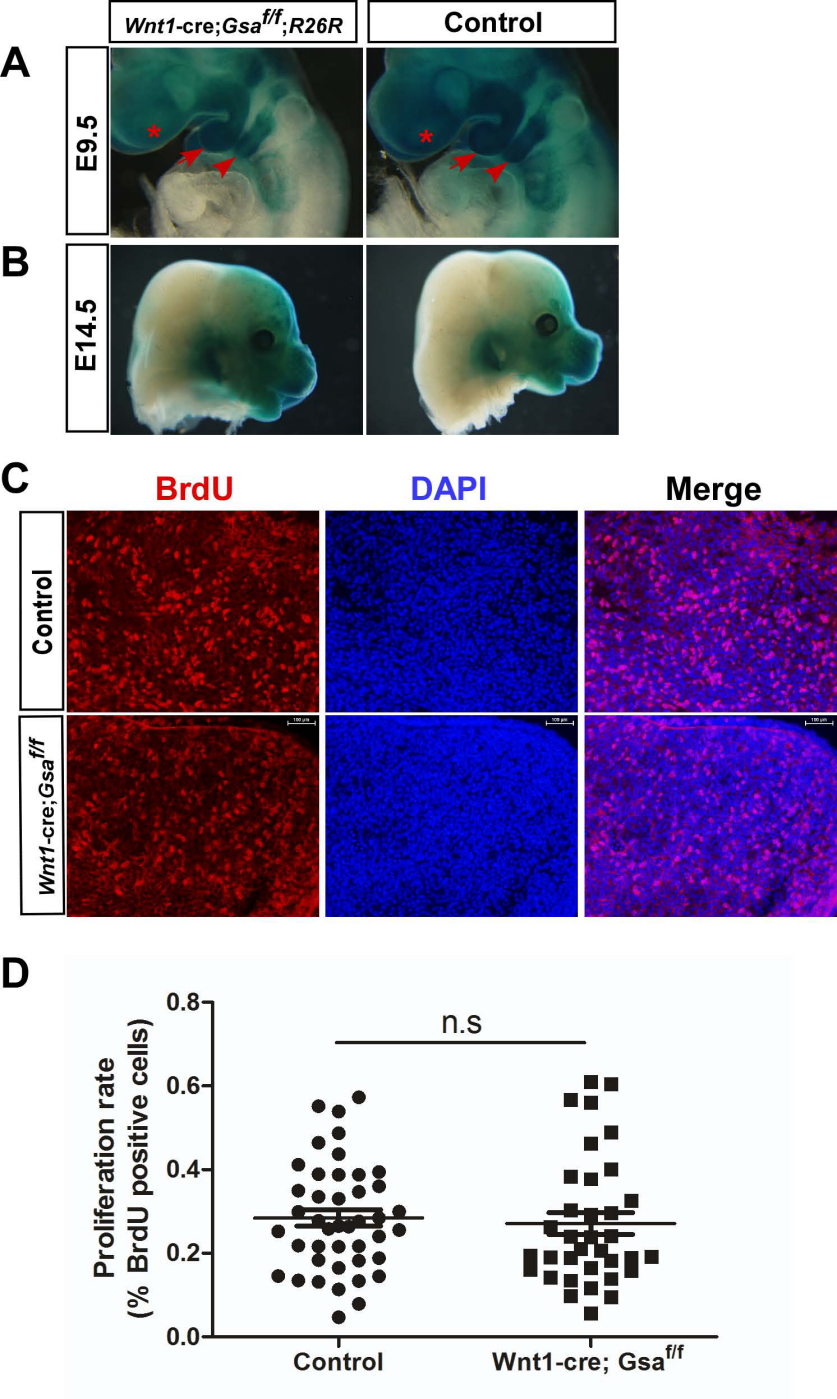
Conditional deletion of *Gsα* in NCCs does not affect neural crest migration or cell proliferation. (A, B) Whole-mount X-gal staining of E9.5 and E14.5 embryos. Normal distribution of migratory neural crest cells in*Wnt1-cre;Gsα*^*f/f*^ mutants. FNP (asterisk), BA1 (arrow) and BA2 (arrowhead). (C, D) Immunofluorescent labeling (C) and quantification (D) of BrdU positive cells in coronal maxilla sections. At least thirty-five sections were randomly selected from six pairs of E13.5 *Wnt1-cre;Gsα*^*f/f*^ mutants and controls. n.s, no significant difference. BA1, first branchial arch; BA2, second branchial arch; FNP, frontonasal prominence.

To figure out the possible cellular mechanisms responsible for craniofacial defects in *Wnt1-cre;Gsα*^*f/f*^ mutant, we first examine whether *Gsα* regulates CNCCs migration. Whole-mount X-gal staining in E9.5 and E14.5 embryos showed a similar CNCCs migration and distribution to frontonasal prominence (FNP), first branchial arch (BA1), and second branchial arch (BA2) between *Wnt1-cre;Gsα*^*f/f*^*;R26R* mutants and controls (Fig 4A and 4B). Next, we examined whether cell proliferation was affected in *Wnt1-cre;Gsα*^*f/f*^ mutants. The CNCCs-derived mesenchymal cell proliferation rate within craniofacial regions, as measured by BrdU incorporation, was identical between E13.5 *Wnt1-cre;Gsα*^*f/f*^ mutants and controls (Fig 4C and 4D). Therefore, these results suggest that specific deletion of *Gsα* in NCCs has no effect on migration or proliferation of CNCCs.

### Specific deletion of *Gsα* in NCCs leads to accelerated osteochondrogenic differentiation

**Fig 5:**
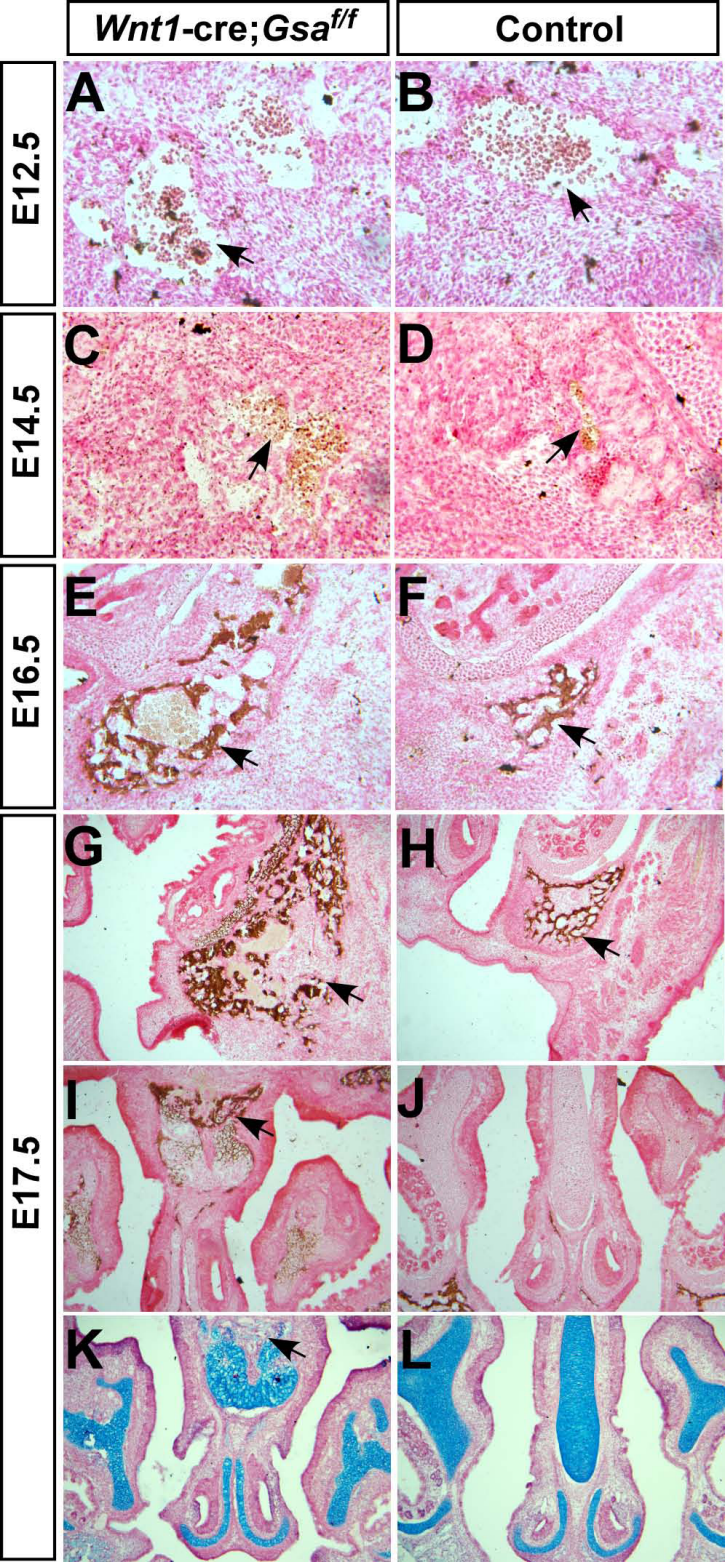
Loss of *Gsα* in NCCs results in abnormal ossification. (A-H) Von Kossa and nuclear red staining of heads coronal sections in *Wnt1-cre;Gsα*^*f/f*^ mutants and controls from different embryonic stages. The aggregated mesenchymal cell in maxilla are similar between E12.5 *Wnt1-cre;Gsα*^*f/f*^ mutant and control (arrows in A and B); however, the ossification region in E14.5 *Wnt1-cre;Gsα*^*f/f*^ mutant is larger than that in control (arrows in C and D), and this phenotype are much more severe at later embryonic stages (arrows in E-H). (I-L) Von Kossa staining (I, J) and alcian blue staining (K, L) show the abnormal ossification and malformation of nasal septum cartilage in E17.5 *Wnt1-cre;Gsα*^*f/f*^ mutants (arrows in I and K).

It has been demonstrated that craniofacial bones are more commonly generated through intramembranous ossification, in which neural crest-derived mesenchymal cells within craniofacial connective tissues aggregate into blastema and then directly differentiate into osteoblasts without cartilage templates [25,26]. Next, we performed Von Kossa staining to assess the ossification process. Histological analysis showed comparable condensation of mesenchymal cells in E12.5 *Wnt1-cre;Gsα*^*f/f*^ mutant and control (Fig 5A and 5B); however, from E14.5 to E17.5, the ossification region was significantly increased in *Wnt1-cre;Gsα*^*f/f*^ mutants compared to controls (Fig 5C–5H). In addition, abnormal ossification and malformed cartilage were observed in the nasal septum of E17.5 *Wnt1-cre;Gsα*^*f/f*^ mutant (Fig 5I–5L).

**Fig 6:**
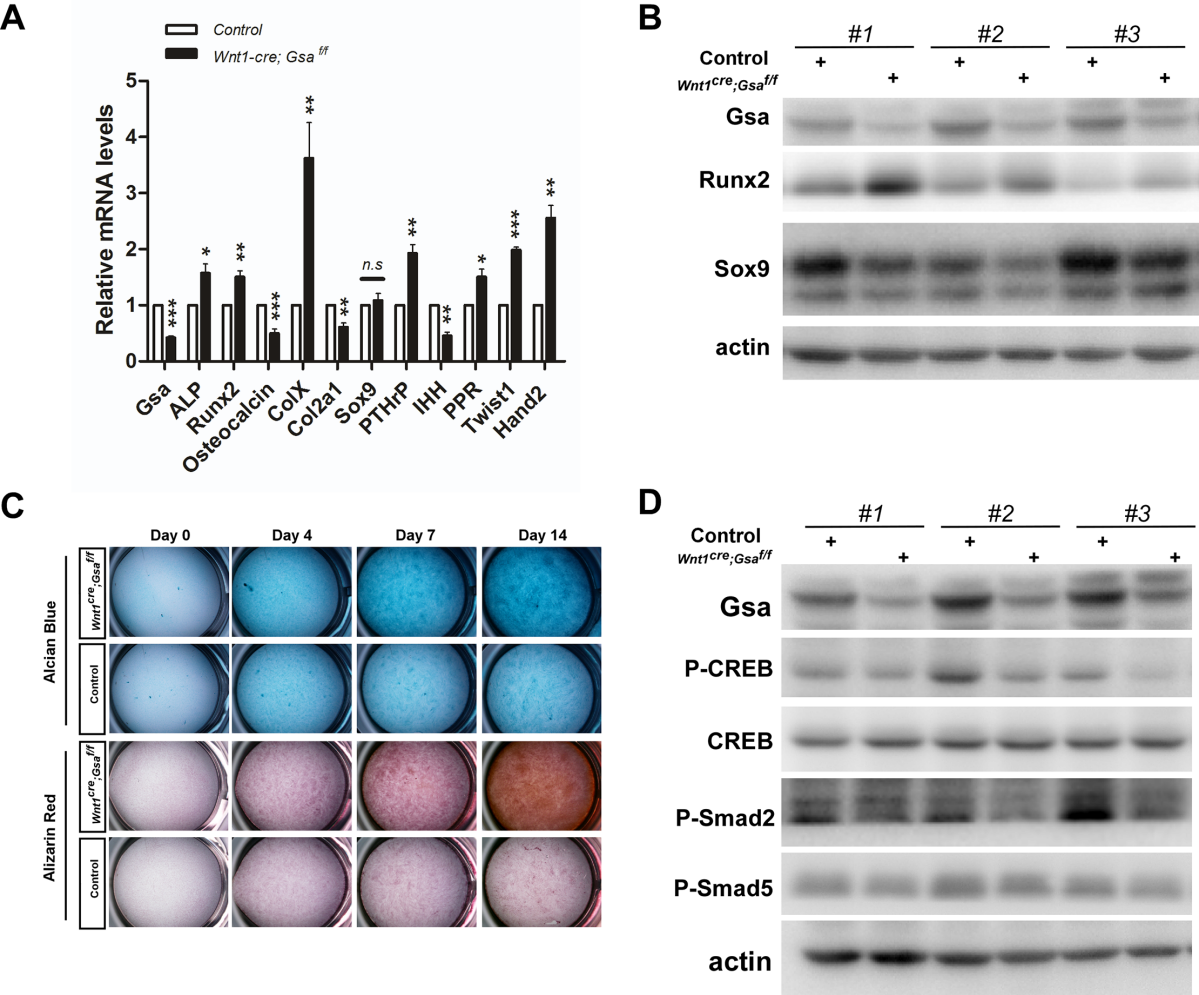
Loss of *Gsα* in NCCs leads to accelerated osteochondrogenic differentiation. (A) RT-qPCR analysis of relative mRNA levels in E16.5 isolated mandible and maxilla tissues. Data shown are normalized ratio of *Wnt1-cre;Gsα*^*f/f*^/Control (mean±SEM); student’s t-test; *p < 0.05; **p <0.01; ***p <0.001; n≥6. (B) Western blot analysis of protein expression of Gsα, Runx2 and Sox9 in E16.5 isolated mandible and maxilla tissues. Data shown are from 3 pairs of mutants and controls. (C) Alcian Blue and Alizarin Red staining show accelerated *in vitro* chondrogenic and osteogenic differentiation in *Wnt1-cre;Gsα*^*f/f*^ mutant cells. Craniofacial mesenchymal cells from *Wnt1-cre;Gsα*^*f/f*^ mutants and controls were subjected to osteochondrogenic differentiation in differentiation medium (complete medium supplemented with 10mM β-glycerophosphate, 50 μg/ml ascorbic acid and 2.5 μM retinoic acid) for indicated days. (D) Western blot analysis of protein expression of Gsα, P-CREB, CREB, P-Smad2 and P-Smad5 in E16.5 isolated mandible and maxilla tissues. Data shown are from 3 pairs of mutants and controls.

Next, to investigate whether the abnormal ossification of mesenchymal cells and nasal cartilage malformation in *Wnt1-cre;Gsα*^*f/f*^ mutants were due to abnormal osteochondrogenic differentiation, we analyzed the expression levels of genes that required for osteochondrogenic differentiation in mandible and maxilla tissues. The mRNA level of *ALP* (an early osteoblastic differentiation marker) and *Runx2* (an osteogenic differentiation transcription factor) were both significantly increased, while *osteocalcin* (a late marker for terminally differentiated osteoblast) was decreased in *Wnt1-cre;Gsα*^*f/f*^ mutants relative to controls (Fig 6A), suggesting that *Gsα* deficiency accelerates osteogenic differentiation but inhibits osteoblast maturation. Although there was no difference in the mRNA level of chondrocytic gene *Sox9* between *Wnt1-cre;Gsα*^*f/f*^ mutants and controls, the expression of *Col2a1* (a marker for proliferative chondrocytes) was decreased, and *ColX* (a marker for differentiated chondrocytes) was markedly increased in *Wnt1-cre;Gsα*^*f/f*^ mutants (Fig 6A), suggesting that *Gsα* deficiency inhibits chondrocytes proliferation but accelerates chondrogenic differentiation. Previous study showed that Indian hedgehog (Ihh) and parathyroid hormone related protein (PTHrP) signaling form a negative feedback loop to maintain chondrocyte proliferation and inhibit hypertrophic differentiation [27,28,29,30,31]. It has been reported that *Gsα* negatively regulates chondrocyte differentiation and is the major mediator of PTHrP signaling [19]. To further explain the effect of *Gsα* deficiency in chondrocytes, we analyzed the expression of *PTHrP*, *PPR* (receptor for PTHrP) and *Ihh*. Results showed that *PTHrP* and PPR were up-regulated, and *Ihh* was down-regulated in *Wnt1-cre;Gsα*^*f/f*^ mutants (Fig 6A), suggesting that loss of *Gsα* affected the Ihh-PTHrP signaling. Hand2 and Twist1 are known regulators of Runx2 [32,33], we found the expression of these two factors were significantly up-regulated in *Wnt1-cre;Gsα*^*f/f*^ mutants. Furthermore, the protein expression of Runx2 and Sox9 was confirmed by western blot, results revealed that Runx2 was up-regulated and Sox9 was down-regulated in the craniofacial tissues of *Wnt1-cre;Gsα*^*f/f*^ mutants (Fig 6B). Together these results suggest that osteochondrogenic differentiation is accelerated in *Wnt1-cre;Gsα*^*f/f*^ mutants.

Furthermore, to examine whether *Gsα* directly regulates osteochondrogenic differentiation, primary craniofacial mesenchymal cells were cultured and induced to initiate osteochondrogenic differentiation in medium with β-glycerophosphate and ascorbic acid [34,35]. The chondrogenic and osteogenic differentiation were assessed at indicated days by Alcian Blue and Alizarin Red staining respectively. As shown in Fig 6C, *Gsα*-deficient cells exhibited more cartilage matrix depositions and mineralized matrix depositions than control cells. Interestingly, we found that from induced day 7 almost all *Gsα*-deficient cells accumulated gradually to form a cell mass, and these cell mass showed extensive Alcian Blue or Alizarin Red staining, but none in control cells (S3 Fig, 8 out of 9 in *Wnt1-cre;Gsα*^*f/f*^ mutants, none in 10 controls). This *in vitro* culture process resembled the mesenchymal condensation *in vivo*, suggesting that osteochondrogenic differentiation is accelerated in *Wnt1-cre;Gsα*^*f/f*^ mutants. Taken together, our data indicate that specific deletion of *Gsα* in NCCs leads to accelerated osteochondrogenic differentiation.

The cAMP response element binding protein (CREB) mediates-PTH signaling and bone morphogenetic protein (BMP) signaling have been demonstrated to regulate osteoblast differentiation and bone formation [36,37,38,39,40,41]. To determine whether these signaling pathways were affected by Gsα deficiency, we examined the expression of downstream protein in dissected mandible and maxilla tissues by immunoblotting. In *Wnt1-cre;Gsα*^*f/f*^ mutants, the phosphorylation of CREB (P-CREB), and the phosphorylation of smad2 and smad5 (P-Smad2 and P-Smad5), were down-regulated (Fig 6D). These results suggest that loss of *Gsα* in NCCs leads to impaired CREB and BMP signaling.

## Discussion

It has been suggested that *Gsα* signaling plays an important role in craniofacial development. Sakamoto *et al*. found that specific deletion of *Gsα* in differentiated osteoblasts using collagen Iα1-cre resulted in hypoplastic craniofacial bones and a 58% penetrance of cleft palate phenotype [21], and chondrocyte-specific *Gsα* ablation mice exhibited cyanosis and domed skull [19]. In our study, we generate neural crest cells-specific *Gsα* KO mice by using *Wnt1*^*cre*^-LoxP system and investigate the role of *Gsα* in craniofacial development. *Wnt1-cre;Gsα*^*f/f*^ mutants are viable at birth but quickly become cyanotic and have feeding difficulties. All mutants die within hours after birth and exhibit dramatic craniofacial deficiencies including domed skull, malformed and shortened maxilla and mandible, cleft palate and craniofacial skeleton defects. Further evidence indicates that accelerated osteochondrogenic differentiation lead to the craniofacial skeletons defects, and consequently cause the cleft palate in *Wnt1-cre;Gsα*^*f/f*^ mutant. Taken together, our study demonstrates that Gsα is required for cranial neural crest cells-derived craniofacial development.

The *GNAS* gene mutations have been implicated in multiple human diseases. The activating *Gsα* mutations lead to endocrine tumors, McCune–Albright syndrome (MAS), and fibrous dysplasia of bone; the heterozygous loss-of-function in *GNAS* cause Albright hereditary osteodystrophy (AHO), a syndrome characterized with short stature, brachydactyly, developmental delay or mental deficits, facial defects such as orbital hypertelorism and depressed nasal bridge. The maternal inheritance of *Gsα* mutations cause AHO with multihormone resistance [termed pseudohypoparathyroidism type IA (PHPIA)], and the paternal inheritance of *Gsα* mutations only lead to AHO phenotype [termed pseudopseudohypoparathyroidism (PPHP)] [11,17]. Recently, a research group reported that a 6-month-old patient with PHPIA caused by *GNAS* mutation had round face and craniosynostosis, which is characterized by synostosis of the coronal, frontal, and sagittal sutures [42]. Interestingly, *Wnt1-cre;Gsα*^*f/f*^ mutants show round face (Fig 1K), shortened snout (Fig 1G, 1I and 1K), hypertelorism (Fig 1G) and craniosynostosis (Fig 2A), these phenotypes are highly similar to the clinical phenotypes observed in *GNAS* mutations diseases.

During the development of mouse embryos, the formation of palate contains multistep, including extension, elevation and fusion of palatal shelves. In detail, palatal shelves arise from maxillary prominence at E12.5 and grow vertically down to the two sides of tongue at E13.5; subsequently, the bilateral shelves elevate horizontally and meet at the midline, then followed by the fusion occurred between E14.5 and E15.5 [43,44]. In light of this process, cleft palate could result from intrinsic defects within palate, such as deficiencies in palatal mesenchyme cells proliferation and palatal epithelium fusion, or a secondary consequence of other craniofacial malformations, such as hypoplastic mandible, physical obstruction of protuberant tongue and palatal bone formation defect [45,46,47,48,49,50,51,52]. Since neural crest cells give rise to the majority of mesenchyme cells within palate and craniofacial skeletons, it raises a question that whether the cleft palate in *Wnt1-cre;Gsα*^*f/f*^ mutant results from a primary defect in palatal development or is secondary to craniofacial skeletal defects. We further demonstrate that the palatal shelves from *Wnt1-cre;Gsα*^*f/f*^ mutant retain the ability to fuse *in vitro*, and the deletion of *Gsα* in palatal shelves by *Nestin-cre* does not cause cleft palate (data not shown). Additionally, the protuberant tongue observed in *Wnt1-cre;Gsα*^*f/f*^ mutant may result from abnormal ossification of hyoid bone and consequently lead to physical obstruction between palatal shelves. Collectively, we conclude that *Gsα* signaling is required for the craniofacial skeleton development and the cleft palate in *Wnt1-cre;Gsα*^*f/f*^ mutant is caused by craniofacial skeletal defects.

Endothelin receptors, including Endothelin-A (ET_A_) and Endothelin-B (ET_B_), are G-protein-coupled receptors (GPCR), and signals via ET_A_ and ET_B_ can be transmitted by G-proteins, Gq, Gi, and/or Gs class. Target deletion of ET_A_, or its ligand endothelin-1 (ET-1), or endothelin converting enzyme-1 (ECE-1) causes cleft palate and abnormal craniofacial bones [53,54,55,56,57]. It has been shown that Gαq/Gα11 proteins mediate ET-1 signaling in pharyngeal arch mesenchyme [58], and neural crest-specific deletion of Gαq/Gα11 results in craniofacial defects similar to those observed in mice lacking ET_A_ or ET-1 [59]. Interestingly, inactivation of Gα12/Gα13 in neural crest cells resulted in cardiac malformations but the head morphology is grossly normal. In our study, neural crest-specific deletion of *Gsα* results in craniofacial abnormalities which are different from ET_A,_ ET-1, Gαq/Gα11, or Gα12/Gα13 knockout mice. These results suggest that the activating pathways for *Gsα* signaling in regulating NCC development are different from that for Gαq/Gα11 or Gα12/Gα13 signaling. Previous studies indicate that Hand2 and Twist1 can negatively regulate Runx2 activity and control mandibular ossification, the reducing of Twist1 or Hand2 levels causes the premature ossification and closure of the cranial sutures [32,33,60,61]. Interestingly, premature ossification of mandible and craniosynostosis phenotypes in *Wnt1-cre;Gsα*^*f/f*^ mutants are similar to those observed in *Hand2* and *Twist1* mutant mice. Moreover, *Runx2*, an obligatory ossification factor, is significantly increased in mandible and maxilla tissues of *Wnt1-cre;Gsα*^*f/f*^ mutants. In order to explain this premature ossification phenotype, we examine the expression of *Hand2* and *Twist1*. Intriguingly, these two factors are both increased in the mandible tissues of *Wnt1-cre;Gsα*^*f/f*^ mutants, suggesting that the molecular mechanism for Runx2 upregulation in *Gsα* mutants may be independent of *Hand2* and *Twist1*.

During craniofacial skeleton development, CNCCs populate into FNP and BA regions, then proliferate and differentiate into mesenchymal cells, and ultimately develop into bones and cartilages [62,63,64]. Precise coordination of NCCs migration, proliferation, and differentiation is essential for craniofacial skeleton development. Our data show that NCCs-specific ablation of *Gsα* has no effect on CNCCs migration or cell proliferation (Fig 4), but significantly accelerate the osteochondrogenic differentiation (Figs 5 and 6 and S3 Fig). Previous reports have revealed the function of *Gsα* signaling in regulating chondrocyte and osteoblast differentiation. The activating Gsα mutations inhibit osteoblast differentiation [65,66,67] and the differentiation from proliferating to hypertrophic chondrocytes [68]. Conversely, the inactivating Gsα mutations may promote osteoblast differentiation [11,69] and chondrocyte differentiation within growth plate [70,71,72]. Furthermore, both the *Gnas* KO mice and chondrocyte-specific *Gsα* KO mice show accelerated hypertrophic differentiation of growth plate chondrocytes and upregulation of PTHrP [12,19,73], and ablation of *Gsα* in early osteoblast lineage results in accelerated osteogenic differentiation [20]. Our results show that the expression of *ColX*, a marker for hypertrophic chondrocytes, is significantly increased in *Wnt1-cre;Gsα*^*f/f*^ mutants (Fig 6A), this result is in line with previous evidence that *Gsα* is a negative regulator of chondrocyte differentiation [12,19,73]. It has been reported that Ihh-PTHrP form a negative feedback loop to regulate chondrocyte proliferation and differentiation, by which Ihh activates the expression of PTHrP in periarticular chondrocytes and PTHrP inhibits the expression of Ihh in proliferating chondrocytes [27,28,29,30,31]. Previous study with chondrocytes *in vitro* also suggested PTH/PTHrP receptors may regulate expression of Col X [74]. Our data show that an increase of *PTHrP* and *PPR*, and a decrease of *Ihh* in the craniofacial tissues of *Wnt1-cre;Gsα*^*f/f*^ mutants. These results are consistent with previous data that *Gsα* negatively regulates chondrocyte differentiation and is the major mediator of Ihh-PTHrP signaling. The inactivation of *Gsα* in calvarial osteoblasts resulted in the up-regulation of *ALP* [21], and osteoblast/osteocyte-specific ablation of *Gsα* decreased the expression of *osteocalcin* in skull bone samples [20]. Our results, consistent with these findings, reveal that NCCs-specific *Gsα* deficiency strikingly increases *ALP* and decrease *osteocalcin* (Fig 6A).

What is the molecular mechanism of *Gsα* regulating osteochondrogenic differentiation? Wu et al. reported that *Gsα* deficiency in early osteoblast lineage reduced the commitment of mesenchymal progenitors to osteoblast lineage via suppressing Wnt signaling; in addition, they reported accelerated differentiation of osteoblasts into osteocytes in *Gsα* deficiency mice, but the molecular mechanism of *Gsα* underlying the regulation of this differentiation process remained unclear [20]. The phenotype in *Wnt1-cre;Gsα*^*f/f*^ mutants resembles that in *PTH-/-* mice [71,75,76]. It has been revealed that BMP signaling and CREB mediates PTH signaling both play critical roles in regulating osteoblast differentiation and bone formations [36,37,38,39,40,41]. Recently, a study demonstrated that PTH-CREB signaling pathway functioned as an effective activator of BMP2 expression [77]. Our data show that P-CREB, a downstream transcriptional factor for *Gsα*-cAMP-PKA, and P-Smad2 and P-Smad5, the downstream signaling effectors for TGF-β and BMP respectively, are suppressed by loss of *Gsα* (Fig 6D). These results suggest that *Gsα* regulating osteochondrogenic differentiation may be attributed to CREB and BMP signaling, but the detailed molecular mechanism need to be further deciphered. Since the craniofacial phenotypes exhibit some differences between *Wnt1-cre;Gsα*^*f/f*^ mutants and *PTH-/-* mice, there might exist other possibilities for *Gsα* regulating craniofacial development. Regard *et al*. found that Wnt/β-catenin signaling is regulated by Gsα proteins during both skeletal development and fibrous dysplasia disease [78] and Wnt/β-catenin signaling has been shown to regulate the craniofacial morphogenesis [79,80]. It would be interesting to investigate the underlying relationship of Gsα, Wnt/β-catenin, TGF-β and BMP signaling in craniofacial development.

In conclusion, our results demonstrate that *Gsα* signaling is required for the neural crest cells-derived craniofacial morphogenesis. Conditional deletion of *Gsα* in neural crest cells results in accelerated osteochondrogenic differentiation and eventually leads to craniofacial malformations.

## Materials and Methods

### Animals

All mice were housed in a specific pathogen-free facility, and all experiments were conducted in accordance with the guidelines and under the approval of the Animal Care Committees at Sichuan University. *Gsα*^*flox/flox*^ mice and Wnt1-cre transgenic mice have been described previously [22,23]. *Gsα*^*flox/flox*^ mice were crossed with Wnt1-cre mice to obtain the*Wnt1-cre;Gsα*^*f/+*^ heterozygous, the tissue-specific *Gsα* knockout mice (*Wnt1-cre;Gsα*^*f/f*^) were obtained from the crosses between *Gsα*^*flox/flox*^ mice and *Wnt1-cre;Gsα*^*f/+*^ heterozygous. *Wnt1-cre;Gsα*^*f/+*^ heterozygous had no obvious phenotype. *Gsα*^*flox/flox*^ and *Gsα*^*flox/+*^ were used as controls except where otherwise specified. These mice were genotyped by PCR using genomic DNA isolated from tails, primers were listed as in Table 1.

**Table 1:** 

Gene	Forward primer	Reverse primer
*Wnt1-cre*	CATACCTGGAAAATGCTTCTGTCC	TCCCCAGAAATGCCAGATTACG
*Gsα*	GAGAGCGAGAGGAAGACAGC	TCGGGCCTCTGGCGGAGCTT

### Skeleton staining

The skeletal preparation and Alizarin Red and Alcian Blue staining were performed according to standard techniques with minor modifications [81]. Briefly, the embryos or neonates were de-skinned and fixed in 100% ethanol overnight, then followed by 100% acetone to remove fat. The skeleton was stained in Alcian Blue solution (150mg/L Alcian Blue 8 GX, 80% ethanol, 20% acetic acid) for two days at 37°C, then washed in 95% ethanol for 8–12 hours and cleared in 2% KOH solution for 24 hours, followed by staining in Alizarin Red solution (50mg/L Alizarin Red in 2% KOH) for 24 hours. Finally, the skeletons were cleared in water and stored in 20% glycerol.

### Histological analysis

Mouse embryos were fixed in 4% paraformaldehyde, washed and cryoprotected in 30% sucrose then embedded in OCT (Tissue-tek), and sectioned at 14 μm. Haematoxylin and eosin (H&E) staining, Von Kossa staining and nuclear red staining were performed following standard procedures.

### Whole-mount X-Gal staining

β-galactosidase expression was detected by X-Gal staining. Embryos were fixed in 4% paraformaldehyde, the staining procedure was performed using the Senescence β-Galactosidase Staining Kit (Beyotime), following the manufacturer’s recommendations.

### Whole-mount immunohistological staining

Whole-mount immunohistological staining was performed as previously described [82]. Anti-2H3 antibody (Hybridoma Bank) was used at a concentration of 1:50.

### Cell proliferation assay

To detect proliferating cells, pregnant mice were injected intraperitoneally with BrdU (5-bromo-2’-deoxy-uridine, 50 μg/g body weight) for 1.5 hours before harvesting embryos. Frozen sections were treated with 2N HCl at 37°C for 20 min then incubated with anti-BrdU antibody (Santa Cruz, #sc32323, 1:200) and DAPI. BrdU positive and DAPI positive cells were automatically counting using Image Pro Plus, and percentage of BrdU^+^ / DAPI^+^ cells was used as proliferation rate.

### Palatal shelves culture

Palatal shelves culture was performed as previously described [83,84,85]. Briefly, palatal shelves were dissected from E13.5 embryos and placed on Millipore filters keeping the two segments touching at the medial edge, cultured for 72 hours and fixed in 4% paraformaldehyde then followed by histological H&E staining.

### Cell culture

Primary craniofacial mesenchymal cell culture and osteochondrogenic differentiation were performed as previously described with minor modification [35,86]. In brief, maxilla was dissected from E13.5 embryos and digested with 0.25% trypsin at 37°C for 30 min, then pipette thoroughly in DMEM containing 10% FBS. Cells were initially seeded at a density of 3 x 10^4^ cell/cm^2^ and cultured in complete medium (DMEM containing 10% FBS supplemented with penicillin, streptomycin, L-glutamate and sodium pyruvate). After 24 hours (Day 0), complete medium was replaced by differentiation medium (complete medium supplemented with 10mM β-glycerophosphate, 50 μg/ml ascorbic acid and 2.5 μM retinoic acid) to induce chondrogenic and osteogenic differentiation. Followed by indicating days, cells were fixed and stained with Alcian blue and Alizarin red as previously described [34,35].

### Real-time quantitative PCR

Total RNA was isolated using the RNeasy kit (QIAGEN, #74134), and cDNA was synthesized with the TransScript One-Step gDNA Removal and cDNA Synthesis SuperMix (Transgen, #AT311). Quantitative real-time PCR (qPCR) was performed using SYBR Green PCR Master Mix (Applied Biosystems, #4367659). Expression levels of mRNA were normalized to β-actin, primer sequences used for qPCR were according to previously published results [20,87], listed as in Table 2.

**Table 2:** 

Gene	Forward primer	Reverse primer
*Gsα*	GCAGAAGGACAAGCAGGTCT	CCCTCTCCGTTAAACCCATT
*ALP*	CACGCGATGCAACACCACTCAGG	GCATGTCCCCGGGCTCAAAGA
*Osteocalcin*	CTCACAGATGCCAAGCCCA	CCAAGGTAGCGCCGGAGTCT
*ColX*	TTCTGCTGCTAATGTTCTTGACC	GGGATGAAGTATTGTGTCTTGGG
*Sox9*	TCGGAACTGCCTGGAAACTTC	GAGGGAGGGAAAACAGAGAACG
*Col2a1*	GAGCAGCAAGAGCAAGGAAAAG	CAGTGGACAGTAGACGGAGGAAAG
*Runx2*	ACCAGTCTTACCCCTCCTATCTGAG	GCAGTGTCATCATCTGAAATACGC
*Twist1*	GGACAAGCTGAGCAAGATTCA	CGGAGAAGGCGTAGCTGAG
*Hand2*	GAGAACCCCTACTTCCACGG	GACAGGGCCATACTGTAGTCG
*PTHrP*	TGGAGTGTCCTGGTATTCCTGCTC	CCACCTTGTTGGTTTCCTGAGTTA
*Ihh*	GCTCTGGCTGCGATTCTTCACACG	CAGAGACTCCGCCCATTGACAGCA
*PPR*	GGCAGTACCTTGTCCCGATTACAT	TACAGTCCCTCCACCAGAATCCAG
*β-actin*	TGTGGTGGTGAAGCTGTAGC	GACGACATGGAGAAGATCTGG

### Statistical analysis

Statistical analysis was performed using two-tailed Student’s *t* test. Data are presented as mean±SEM; *P*<0.05 indicated significant difference.

## Supporting Information

S1 Fig The gross morphologies of craniofacial nerve and cardiac outflow tract are normal in *Wnt1-cre;Gsα*^*f/f*^ mutants. (A-D) Whole-mount staining of anti-neurofilament marker 2H3 in E10.5 (A, B) and E12.5 (C, D) embryos. (E, F) Whole-mount X-gal staining of heart in E17.5 *Wnt1-cre;Gsα*^*f/f*^ mutant and control.
(TIF)

S2 Fig Impaired development of DRG and sympathetic ganglia in *Wnt1-cre;Gsα*^*f/f*^*;R26R* mutants. (A-F) Lateral view of whole-mount X-gal staining of dorsal root ganglion in E15.5 (A, B), E16.5 (C, D) and E17.5 (E, F) *Wnt1-cre;Gsα*^*f/f*^ mutants and controls. (G-J) Ventral view of whole-mount X-gal staining of sympathetic ganglion in E15.5 (G, H) and E17.5 (I, J) *Wnt1-cre;Gsα*^*f/f*^ mutants and controls.
(TIF)

S3 Fig Loss of *Gsα* in NCCs leads to accelerated osteochondrogenic differentiation. Alcian Blue and Alizarin Red staining show accelerated *in vitro* chondrogenic and osteogenic differentiation and cell accumulation in *Wnt1-cre;Gsα*^*f/f*^ mutant cells. All pictures are from three independent experiments, *Wnt1-cre;Gsα*^*f/f*^ mutants (n = 9), controls (n = 10).
(TIF)
