# Supplementary figures and images for: G-Protein α-Subunit Gsα Is Required for Craniofacial Morphogenesis
Source: PLoS One. 2016 Feb 9;11(2):e0147535. doi: 10.1371/journal.pone.0147535 (PMC4747491; doi:10.1371/journal.pone.0147535)

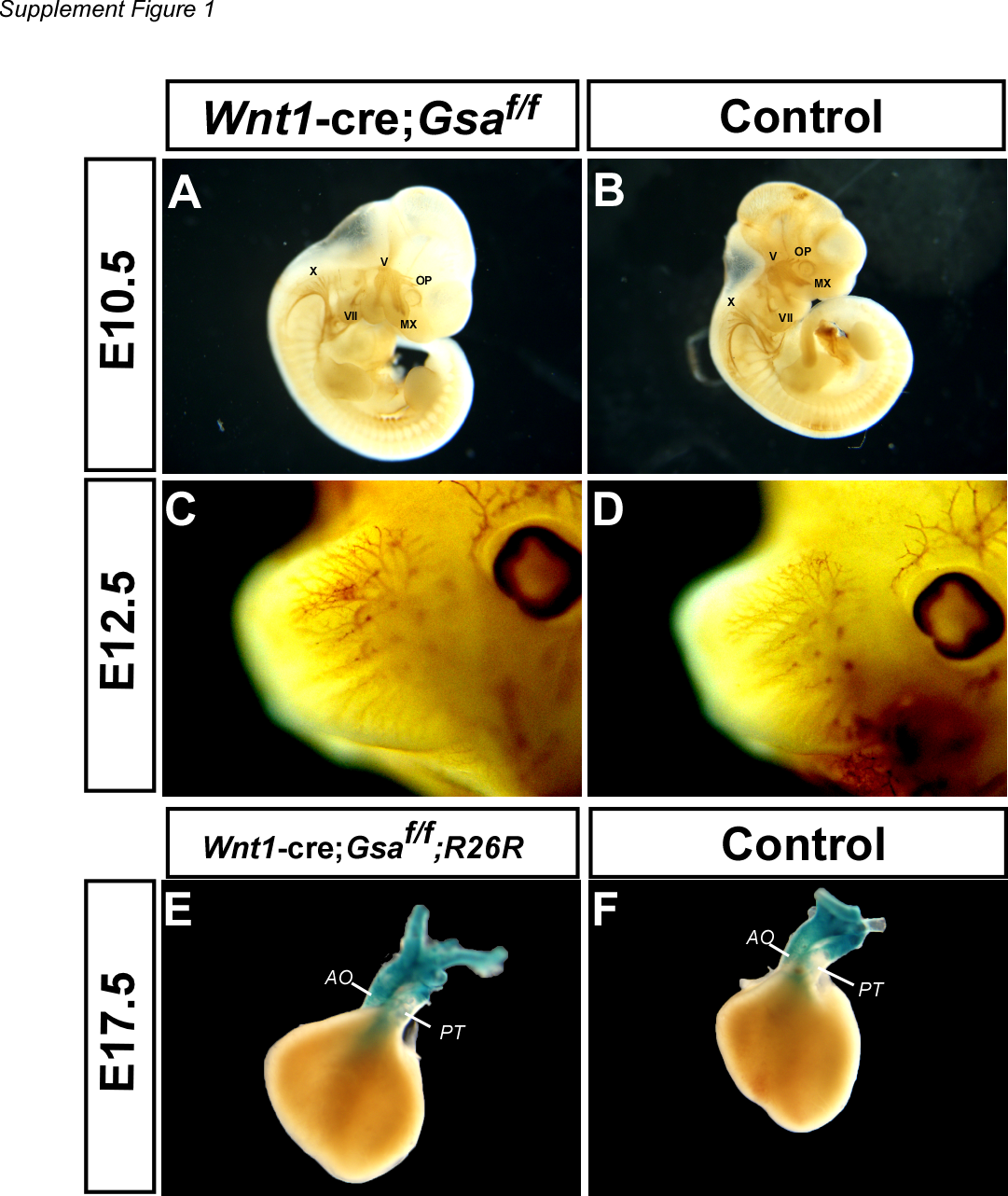

Supplement: S1 Fig — (A-D) Whole-mount staining of anti-neurofilament marker 2H3 in E10.5 (A, B) and E12.5 (C, D) embryos. (E, F) Whole-mount X-gal staining of heart in E17.5 Wnt1-cre;Gsαf/f mutant and control. (TIF) [file pone.0147535.s001.tif]

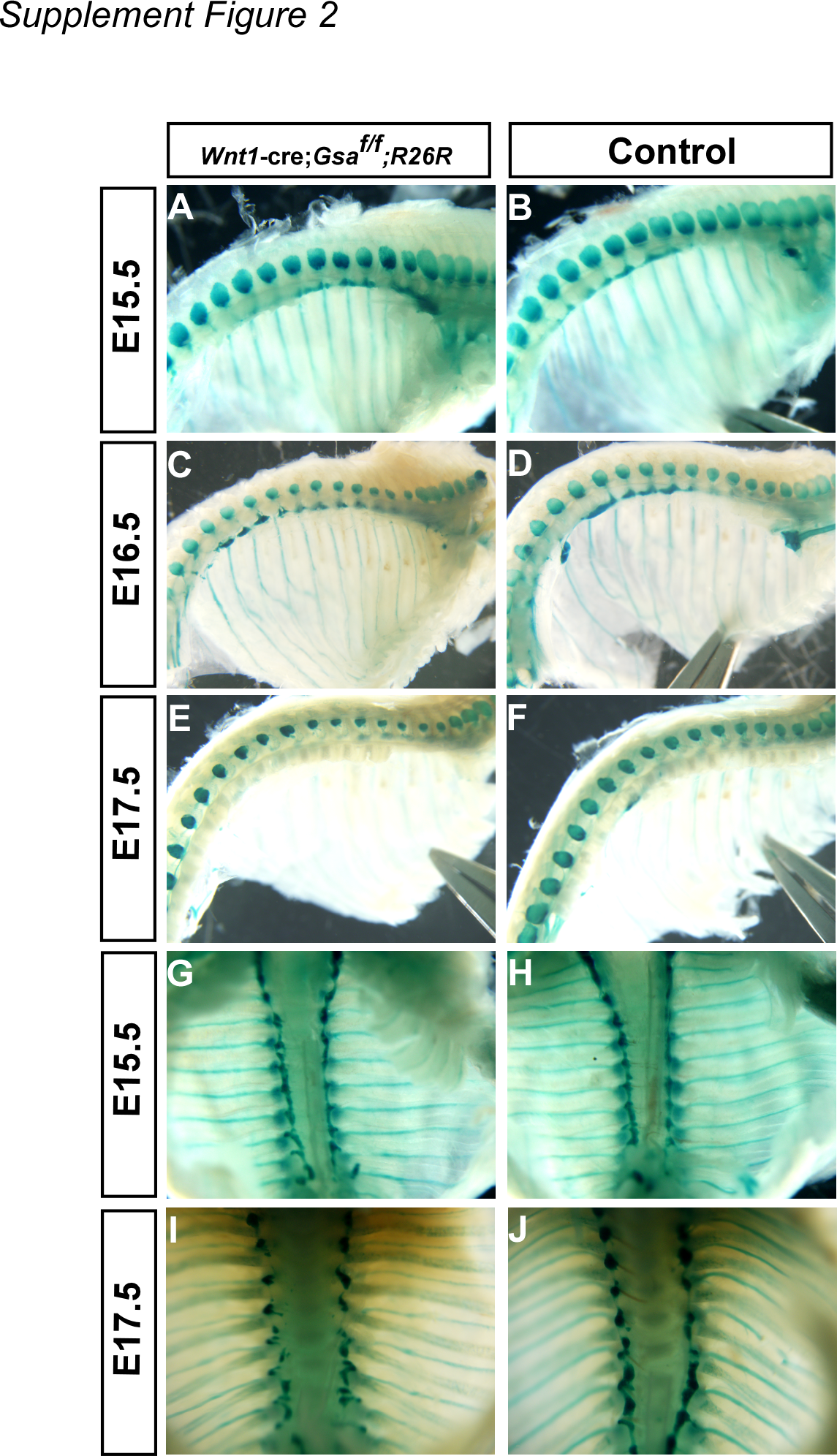

Supplement: S2 Fig — (A-F) Lateral view of whole-mount X-gal staining of dorsal root ganglion in E15.5 (A, B), E16.5 (C, D) and E17.5 (E, F) Wnt1-cre;Gsαf/f mutants and controls. (G-J) Ventral view of whole-mount X-gal staining of sympathetic ganglion in E15.5 (G, H) and E17.5 (I, J) Wnt1-cre;Gsαf/f mutants and controls. (TIF) [file pone.0147535.s002.tif]

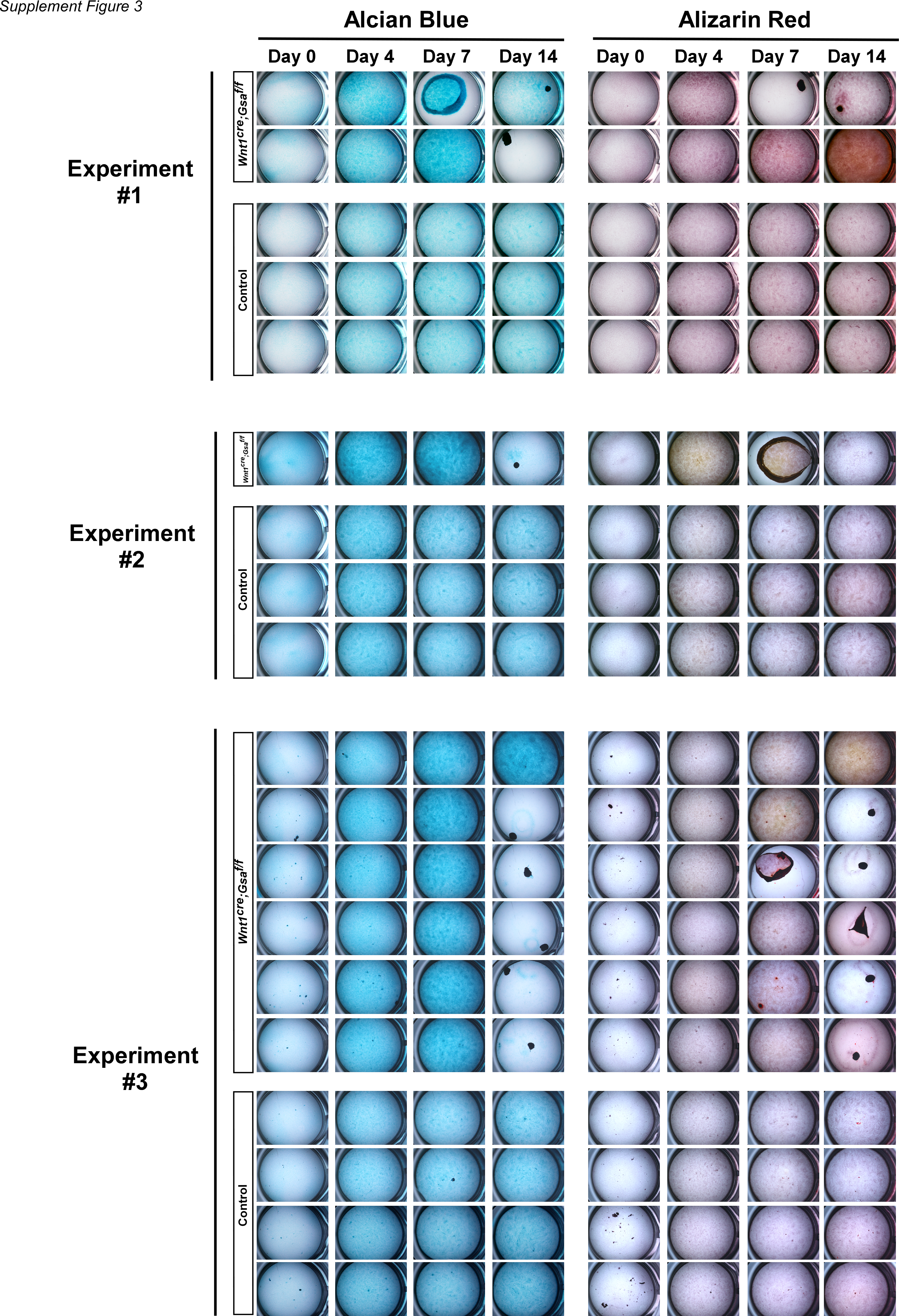

Supplement: S3 Fig — Alcian Blue and Alizarin Red staining show accelerated in vitro chondrogenic and osteogenic differentiation and cell accumulation in Wnt1-cre;Gsαf/f mutant cells. All pictures are from three independent experiments, Wnt1-cre;Gsαf/f mutants (n = 9), controls (n = 10). (TIF) [file pone.0147535.s003.tif]
